# Transcriptome analysis of plasmid-induced genes sheds light on the role of type I IFN as adjuvant in DNA vaccine against infectious salmon anemia virus

**DOI:** 10.1371/journal.pone.0188456

**Published:** 2017-11-21

**Authors:** Mehrdad Sobhkhez, Aleksej Krasnov, Chia Jung Chang, Børre Robertsen

**Affiliations:** 1 Norwegian College of Fishery Science, UiT The Arctic University of Norway, Tromsø, Norway; 2 Nofima AS, Norwegian Institute of Food, Fisheries & Aquaculture Research, Ås, Norway; INRA, FRANCE

## Abstract

A previous study showed that a plasmid expressing IFNa (pIFNa) strongly enhanced protection and antibody production of a DNA vaccine against infectious salmon anemia virus (ISAV) in Atlantic salmon. The vaccine consisted of a plasmid (pHE) expressing the virus hemagglutinin-esterase as an antigen. To increase the understanding of the adjuvant effect of pIFNa, we here compared transcriptome responses in salmon muscle at the injection site at week 1 and 2 after injection of pIFNa, pHE, plasmid control (pcDNA3.3) and PBS, respectively. The results showed that the IFNa plasmid mediates an increase in gene transcripts of at least three major types in the muscle; typical IFN-I induced genes (ISGs), certain chemokines and markers of B- cells, T-cells and antigen-presenting cells. The latter suggests recruitment of cells to the plasmid injection site. Attraction of lymphocytes was likely caused by the induction of chemokines homologous to mammalian CCL5, CCL8, CCL19 and CXCL10. IFN may possibly also co-stimulate activation of lymphocytes as suggested by studies in mammals. A major finding was that both pcDNA3.3 and pHE caused responses similar to pIFNa, but at lower magnitude. Plasmid DNA may thus by itself have adjuvant activity as observed in mammalian models. Notably, pHE had a lower effect on many immune genes including ISGs and chemokines than pcDNA3.3, which suggests an inhibitory effect of HE expression on the immune genes. This hypothesis was supported by an Mx-reporter assay. The present study thus suggests that a main role for pIFNa as adjuvant in the DNA vaccine against ISAV may be to overcome the inhibitory effect of HE- expression on plasmid-induced ISGs and chemokines.

## Introduction

A previous study showed that Atlantic salmon type I interferons (IFNs) possess potent adjuvant effect in combination with a DNA vaccine against infectious salmon anemia virus (ISAV) based on the viral hemagglutinin-esterase (HE) as antigen [1]. Vaccination of salmon with a plasmid expressing HE (pHE) alone gave low antibody titres and a minor protection against ISAV. In contrast, pHE injected together with a plasmid expressing IFNa (pIFNa) gave high levels of IgM antibodies against ISAV and strong protection against ISAV-infection. This demonstrated that type I IFNs enhance adaptive immune responses in Atlantic salmon against the HE antigen of ISAV and thus function as adjuvants. It is known from mammalian studies that recombinant type I IFNs stimulate adaptive immune responses against protein antigens and that the mechanism involves direct stimulation of B-cells, T-cells and dendritic cells (DCs) [2–4]. The protective effects of DNA vaccines against the salmonid rhabdoviruses IHNV and VHSV are also associated with induction of type I IFN stimulated genes (ISGs), but in those studies the effects of antigens could not be dissected from the IFN-inducing activity [5–7]. A benefit of the IFN adjuvanted DNA vaccine against ISAV is that effects of the virus antigen HE and type I IFN can be assessed separately. To increase the understanding of the adjuvant effect of salmon type I IFN, we in the present work did a comparative transcriptome analysis of muscle tissue at the injection site of presmolts at one and two weeks after injection of pIFNa, pHE, plasmid without insert (pcDNA3.3) or PBS. The importance of muscle tissue for the adaptive immune response is underlined by the fact that the adjuvant effect of the IFNa plasmid must be caused by stimulation of immune cells at the muscle injection site since IFNa does not induce antiviral genes systemically in salmon [1, 8]. Moreover, while antigen-presenting cells play a critical role in DNA vaccination of mammals, transfected muscle cells are important for the magnitude and duration of the induced immune response [9]. An essential question in the present work was how pHE influenced expression of immune related genes. Expectedly, pIFNa induced high transcript levels of a multitude of genes including a panel of typical ISGs. In addition, pIFNa strongly enhanced transcripts of B- and T-cell markers suggesting attraction of lymphocytes to the site of plasmid injection. Surprisingly, both pcDNA3.3 and pHE caused similar responses as pIFNa although at a lower magnitude. However, the level of transcripts from several immune genes induced by pHE was lower compared to pcDNA3.3, which suggests that pHE inhibits IFNa signalling. The inhibitory effect of pHE was confirmed by Mx- reporter assay.

## Materials and methods

### Ethics statement

All handling of fish was performed in accordance to the Norwegian “Regulation on Animal Experimentation” and all fish experiments were submitted to and approved by the Norwegian Animal Research Authority (NARA) before initiation.

### Fish

Atlantic salmon (*Salmo salar* L.) presmolts (30–40 g) of the strain Aquagen standard (Aquagen, Kyrksæterøra, Norway) were kept in 300 L tanks supplied with fresh water at 10°C and were fed commercial dry food in Tromsø Aquaculture Research Station, Norway. For injection of plasmids, fish were anesthetized with 0.005% benzocaine (ACD Pharmaceuticals, Norway) and different groups were labeled by tattooing (2% alcian blue, Panjet inoculator). Before harvest, fish were euthanized by an overdose of benzocaine (0.01%).

### Treatment of fish with plasmids

pcDNA3.3 encoding the open reading frame (ORF) of Atlantic salmon IFNa1 (pIFNa) and pcDNA3.3 plasmid without insert were available from a previous study [10]. pcDNA3.3 encoding hemagglutinin-esterase from infectious salmon anemia virus was obtained by subcloning from a construct used previously and is named pHE [1]. The plasmids were purified by EndoFree plasmid purification kit from Qiagen.

Presmolt salmon were injected intramuscularly (i.m.) approximately 1 cm below the dorsal fin with 15 μg plasmids in 50 μl sterile phosphate-buffered saline (PBS) at pH 7.4 or with PBS only. Fish groups (N = 5) included non-treated fish (Group 1) and fish injected with PBS (Group 2), pcDNA3.3 (Group 3), pIFNa (Group 4) and pHE (Group 5). Muscle at the injection site was sampled at one week (W1) and two weeks (W2) after injection and used for RNA isolation.

### Gene expression analysis by reverse transcription quantitative PCR (qPCR)

Muscle samples from the injection site were homogenized using TissueLyser II (QIAGEN) (30 sec, 30 1/s) and total RNA was extracted using the RNeasy® Mini Kit (Qiagen). RNA (500 ng in a 20 μl reaction) was reverse transcribed using high capacity cDNA Reverse Transcription kit (Applied Biosystems). cDNA was diluted 10 times and 5 μl of cDNA was used per 20 μl PCR reaction, which also included primers for target gene (0.25 μM) and Fast SYBR® Green Master Mix (Applied Biosystems). The samples were applied in duplicates and the measurement was done using ABI Prism 7500 FAST Cycler from Applied Biosystems (initial denaturation 95°C: 20s and 40 cycles of 95°C: 3s, and 60°C: 30s). Primer sequences are included in S1 Table. Expression of each gene was calculated relative to Elongation factor1αβ (EF1αβ) as0020described [11]. In S2 Table, expression values were normalized against the levels of EF1αβ and fold change in expression of different genes was calculated against that of PBS group using the method described by Pfaffl [12]. Unpaired t-test with two-tail distribution was used for statistical analysis, *p* ≤ 0.05.

### Microarray analyses

Nofima’s Atlantic salmon oligonucleotide microarray SIQ-6 (GEO Accession no. GPL16555) was produced by Agilent Technologies in the 15 K x 8 format, all reagents and equipment were from the same source [13]. Analyses included five groups, four individuals per time-point; totally forty arrays were used. Total RNA (200 ng per reaction) was labelled with Cy3 using Low Input Quick Amp Labeling Kit and fragmented with Gene Expression Hybridization Kit. Hybridization was performed for 17 hours in an oven at 65°C at rotation speed of 10 rounds per minute. Arrays were washed for one minute with Gene Expression Wash Buffer I at room temperature, and one minute with Gene Expression Wash Buffer II at 37°C and scanned. Data analyses were carried out with Nofima’s bioinformatics package [13]. Global Normalization was performed by equalizing the mean intensities of all microarrays. Next, the individual values for each feature were divided to the mean value of all samples producing expression ratios (ER). The log_2_-ER were calculated and normalized with the locally weighted non-linear regression (Lowess). The data are presented as ratios to PBS injected control plasmid injected groups. Differentially expressed genes were selected by criteria: p < 0.05 and log_2_-ER > |0.8| (1.74-fold). The microarray data presented in this publication has been deposited in the NCBI´s Gene Expression Omnibus (GEO, https://www.ncbi.nlm.nih.gov/geo/) and is available under the accession number GSE106111.

### Reporter gene assay

The reporter plasmid pGL3-Basic-PrMx1 containing the rainbow trout Mx1 promoter fused to the firefly luciferase gene was obtained from Dr. Bertrand Collet, FRS Marine Laboratory, Aberdeen [14]. Renilla luciferase construct was purchased from Sabiosciences. A plasmid expressing EGFP (pEGFP) was obtained from Invitrogen and a plasmid expressing ovalbumin (pOVA) was constructed with the open reading frame of the chicken ovalbumin gene (Acc. Nr. NP_990483) inserted downstream of CMV promoter in the pcDNA3.3 vector. CHSE-214 cells were seeded in 48-well plate (80,000 cells/well) and chemically transfected (Lipofectamin 2000, Thermo Fisher) with 250 ng/well of either pcDNA3.3, pHE, pEGFP or pOVA in combination with 90 ng/well constitutively expressing Renilla luciferase construct (SABiosciences) and 250 ng/well of pGL3-Basic-PrMx1. Cells were stimulated for 24 hours with 1000 U/ml salmon IFNa or left un-stimulated. Twenty four hours post stimulation cells were lysed in 20 μl lysis buffer and 10 μl was used for the luciferase luminescence assays using the Dual-Luciferase® Reporter Assay System (Promega). The luminescence from both firefly and Renilla luciferase was measured using a plate luminometer, Luminoskan Ascent (Thermo Scientific, Beverly, MA), and result are presented as Relative Light Units. Each experiment was set up in six parallels, three wells were stimulated with IFNa and three wells were left un-stimulated. Unpaired t-test with two-tail distribution was used for statistical analysis, *p* ≤ 0.05.

## Results

### Summary of transcriptome responses

**Fig 1:**
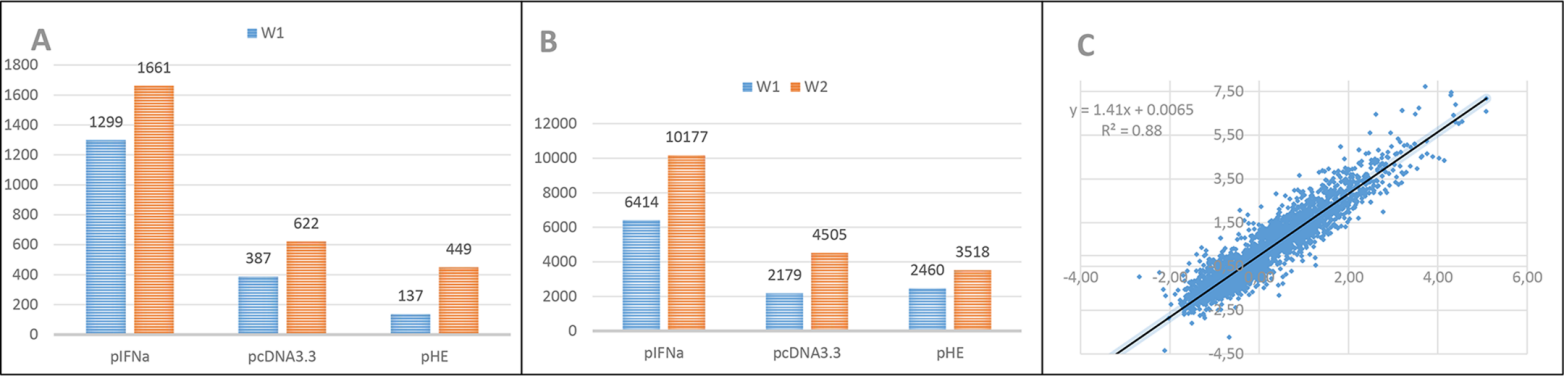
An overview of transcriptome responses to plasmids at the injection sites of skeletal muscles. A: numbers of differentially expressed genes (DEG). B: magnitude of differentially expression assessed as sum of log_2_ (expression ratio-ER) to PBS injected fish for 3670 DEG. C: relationship of transcriptome responses to pIFNa (y-axis) and pcDNA3.3 (x-axis). Data are log_2_(ER) at W2.

The number of differentially expressed genes (DEG) increased from week 1 (W1) to week 2 (W2) in all the three groups of fish injected with plasmid (Fig 1A). The magnitude of responses was consistently greatest in pIFNa group (Fig 1B). Gene expression changes in response to the control plasmid and pIFNa were highly correlated being greater in the latter group (Fig 1C). The virus protein HE modulated responses to plasmid: 98 genes showed difference between pcDNA3.3 and pHE and 61 of these had lower expression in pHE group. A significant fraction of DEGs is involved in immune responses and most of them were up-regulated by all three plasmids.

### Genes involved in innate antiviral immunity

#### IFNs

**Fig 2:**
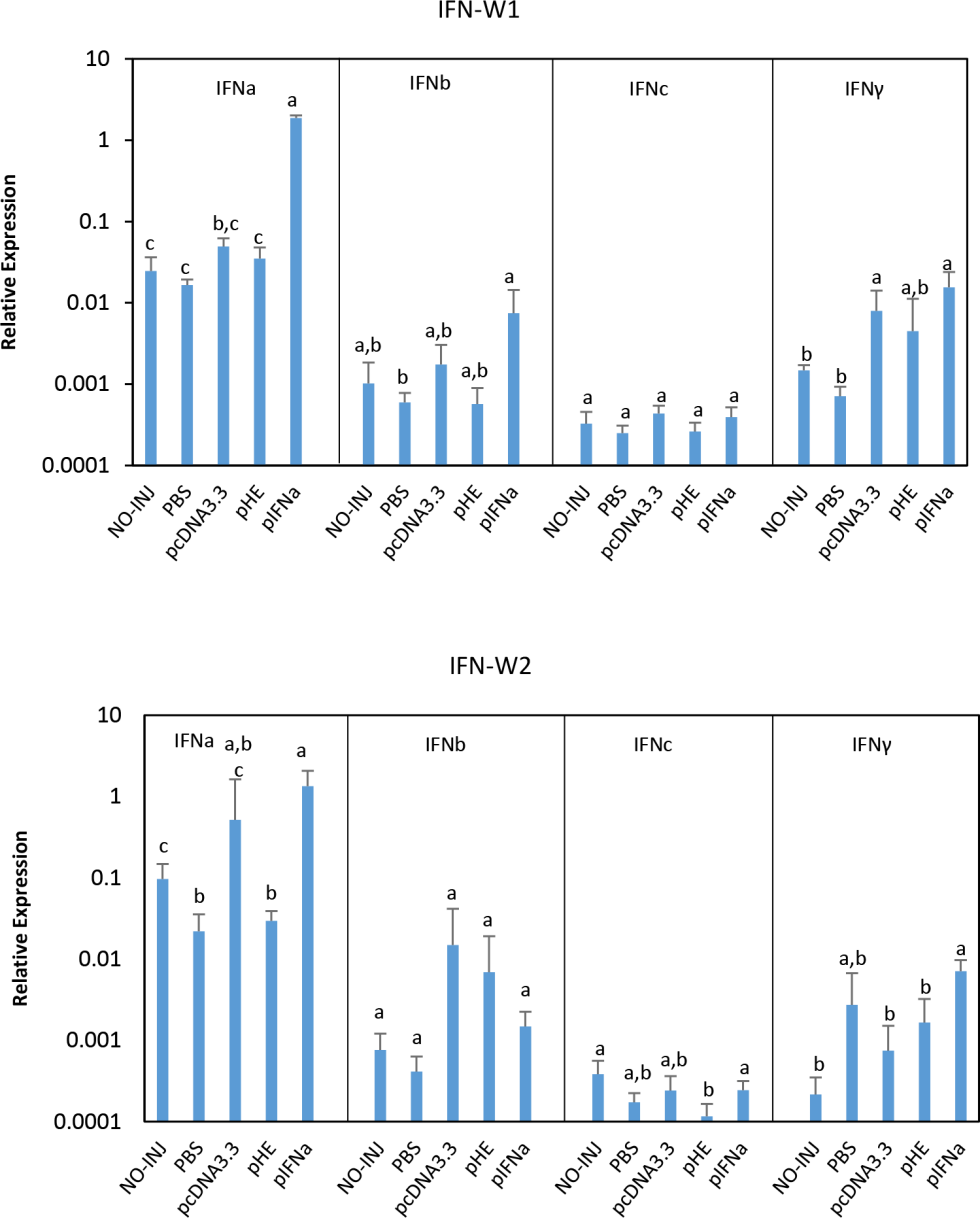
Expression of Type I and Type II IFN in response to plasmids at the injection site. Groups of fish (N = 5) were injected with PBS, pcDNA3.3, pHE or pIFNa, or kept non-treated (NO-INJ). RNA was isolated from muscle at the injection site at one (W1) and two (W2) weeks post injection and expression of different IFN genes was measured by qPCR. Data are presented as mean gene expression relative to expression of EF1αβ +/- SD. Bars not sharing common letter are significantly different (p ≤ 0.05).

Expression of IFNa, IFNb, IFNc and IFN-gamma (IFNg) in muscle were studied by qPCR (Fig 2). Expression of all genes are compared to PBS unless stated otherwise.

Compared to non-treated fish (NO-INJ), injection of PBS caused a minor, but non-significant down-regulation of these genes, except for IFNg at W2. As expected, IFNa expression in fish injected with pIFNa was highest (mean fold increase of 114 times at W1 and 72 times at W2). IFNa transcripts showed an increase in fish injected with pcDNA3.3 at W1 and a larger increase at W2, but the differences were not statistically significant. pHE gave a small increase in IFNa transcripts at W1 and no increase at W2. Expression of IFNb was significantly up-regulated only by pIFNa at W1. The plasmids did apparently not have any effect on IFNc expression. IFNg expression was significantly enhanced by pIFNa at W1, but not at W2. pcDNA3.3 gave significantly increased IFNg expression at W1, but decreased expression at W2. pHE did not have any significant effect on IFNg expression at any time point. Compared to pcDNA3.3, pHE showed lower expression of IFNa, IFNb, IFNc and IFNg, but the differences between the two groups were not significant.

#### Interferon stimulated genes (ISGs)

**Fig 3:**
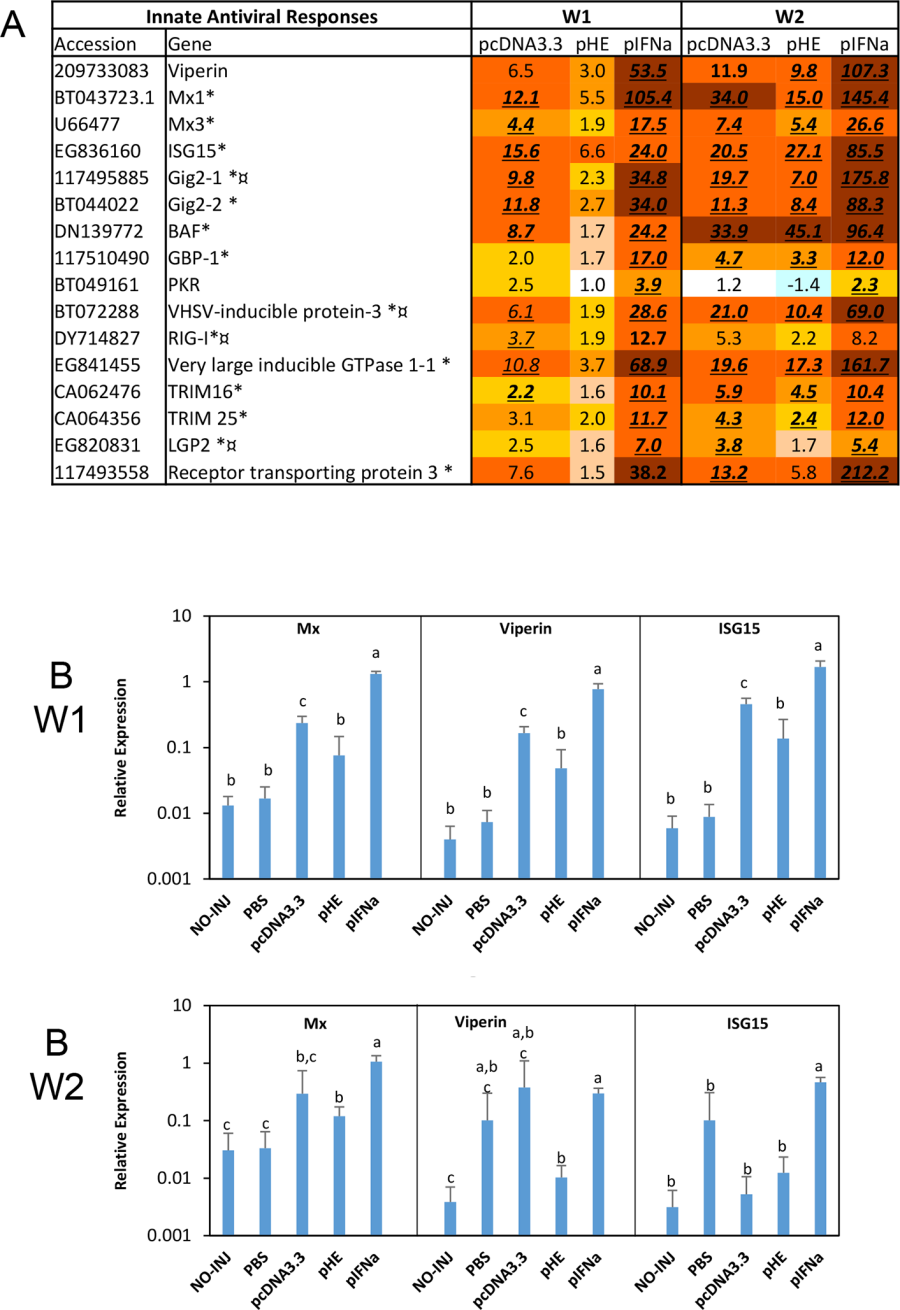
Expression of genes involved in innate antiviral responses. A. Microarray data of IFN stimulated genes (ISGs). Numbers represent fold changes compared to the PBS group in this and subsequent microarray figures, and expression difference (> 1.74-fold, p < 0.06) is indicated with underlined italics. Symbols * and ¤ after gene names denote difference (same criteria) respectively between pIFNa and pcDNA3.3 and between pcDNA3.3 and pHE. B. qPCR of selected antiviral ISGs. The primers for Mx recognize both salmon Mx1 and Mx3. Groups of fish (N = 5) were injected with PBS, pcDNA3.3, pHE or pIFNa, or kept non-treated (NO-INJ). Data are presented as mean gene expression relative to expression of EF1αβ +/- SD. Bars not sharing common letter are significantly different (p ≤ 0.05).

Many genes revealed up-regulated by the plasmids with microarrays and qPCR were previously identified as ISGs in Atlantic salmon TO cells [15] and as virus responsive genes (VRGs) in a transcriptome survey of salmon infected with different RNA viruses [16], totally 125 features. ISGs have crucial roles in innate antiviral immunity. Several ISGs such as Mx, ISG15 and viperin, possess direct antiviral activity, some are involved in virus recognition (RIG-I, LGP2) and some are transcription factors involved in IFN induction or IFN signalling (IRFs, STATs), while the functions of others are less known. As expected, pIFNa caused strong up-regulation of antiviral ISGs both in the microarray (Fig 3A) and qPCR analyses (Fig 3B). The microarray analyses showed that all three plasmids induced multiple other genes with various functions. In all cases the responses were strongest with pIFNa and in most cases weakest with pHE. The strongest responses were detected for Very large inducible GTPase, TRIM16 and 25 as well as Receptor transporting protein 3 and RIG-I like receptor LGP2. The overall response to pIFNa detected by microarray analysis was similar or higher at W2 compared to W1. pcDNA3.3 also up-regulated these genes, but at a substantially lower level than pIFNa. The response to pHE was lower compared to pcDNA3.3 at W1, but the difference had levelled off at W2.

IRFs and STATs have important roles in induction of IFN and/or ISGs. The microarray analysis showed highest up-regulation of IRF1, IRF2, IRF3, IRF5, IRF7 and IRF8 by pIFNa, lower for pcDNA3.3, and in most cases lowest for pHE (Panel A in S1 Fig). Similar profiles were seen for STAT1, STAT2 and STAT3. In contrast, STAT3-like protein and STAT5b were down-regulated by all three plasmids at both W1 and W2 while IRF7 was down-regulated by pHE at W2. As with ISGs, pIFN caused highest responses and pHE the lowest. Similar patterns of expression were observed by qPCR analysis (Panel B in S1 Fig). Expression of IRF1, IRF3, IRF7 as well as STAT1 and STAT2 were significantly up-regulated by pIFNa. Overall, pcDNA3.3 showed a higher stimulatory effect than pHE at both time points, which was statistically significant for IRF3, IRF7, STAT1 and STAT2 at W1.

### Genes involved in inflammation

**Fig 4:**
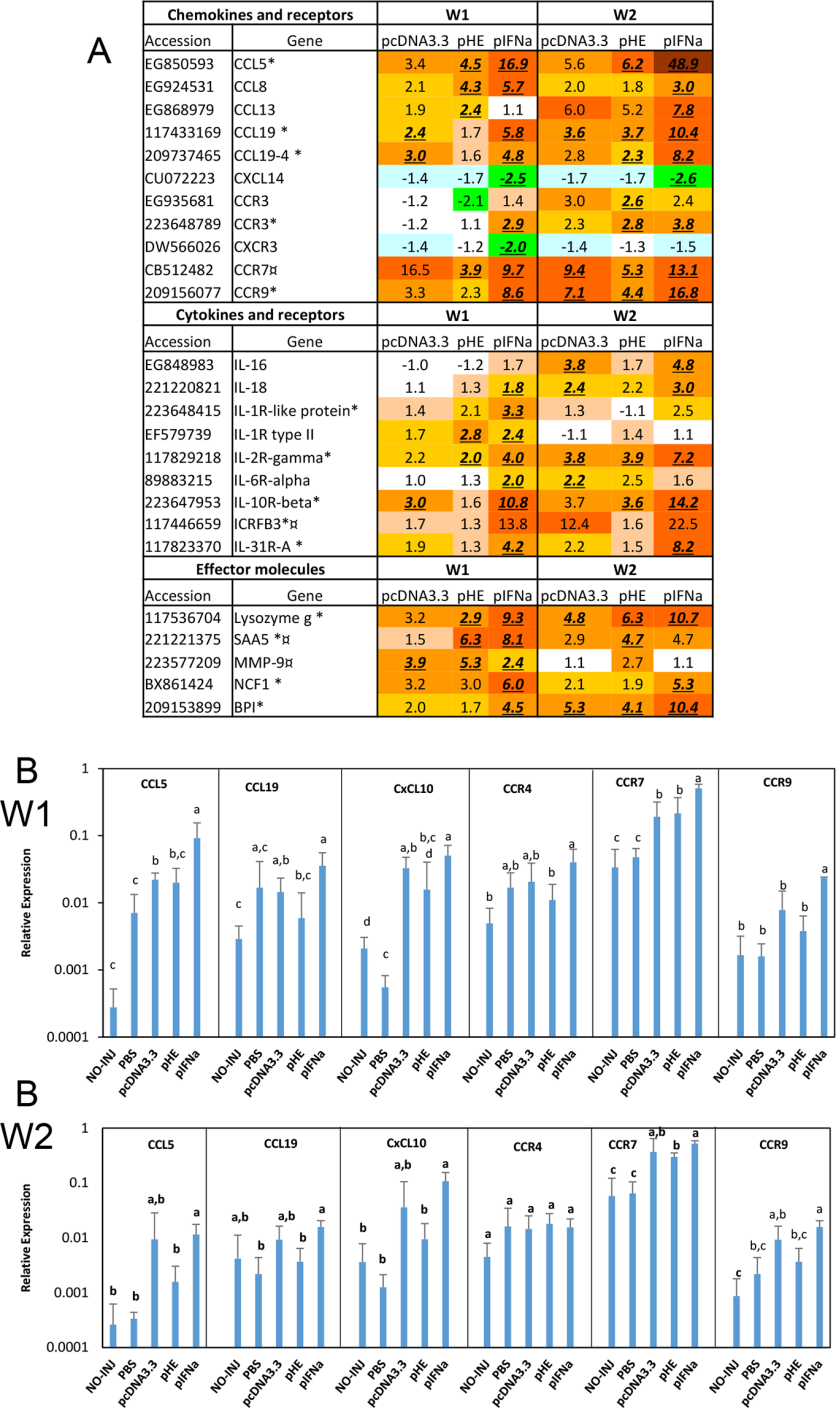
Inflammatory responses. A. Microarray data of chemokines, cytokines and their receptors and effectors. Data produced and presented as for Fig 3. B. Expression of chemokines in response to plasmids measured by qPCR. Treatment groups and sampling for RNA extraction as described in Fig 3. Data are presented as mean gene expression relative to expression of EF1αβ +/- SD, bars not sharing common letter are significantly different p ≤ 0.05).

Injection of plasmids caused a strong inflammation, which was observed as red coloration at the injection site. The microarray data showed that expression of several pro-inflammatory chemokines and chemokine receptors were significantly influenced by plasmid injection (Fig 4A). All three plasmids stimulated expression of one CCL5-like gene, one CCL8-like gene, and two CCL19-like genes. In contrast, a CXCL14-like gene was down-regulated by all three plasmids. pIFNa had the strongest stimulatory effect on the chemokines and most prominently enhanced transcription of the CCL5-like gene at both time points. For CCL5 and most of the other genes, the response was strongest at W2 in the pIFNa group. Of chemokine receptor genes, the plasmids enhanced expression of two CCR3-like genes, one CCR7-like, one CCR9-like and one CXCR3-like gene. Similar to chemokines, pIFNa showed the strongest effect with the exception of CCR7, which had higher expression in response to pcDNA3.3 at W1. pcDNA3.3 and pHE showed small differences in stimulatory effect on chemokines and receptors except that pcDNA3.3 caused stronger enhancement of CCR7-like transcripts at both W1 and W2. The microarray data were supported by qPCR analysis for the CCL5-, CCL19- CXCL10- and CCR9-like genes despite large individual differences (Fig 4B). Among interleukins, only IL-16 and IL-18 were up-regulated by the plasmids and the responses were modest. On the other hand, the microarray data showed that several cytokine receptors were strongly up-regulated by pIFNa at both W1 and W2 with IL10Rb and CRFB3 showing the strongest responses (Fig 4A). These receptors were also up-regulated by pcDNA3.3 and pHE to similar levels except CRFB3, which at W2 had lower expression with pHE compared to pcDNA3.3. The microarray data showed that pHE induced more strongly MMP9 and SAA5 than pcDNA3.3, two inflammatory effectors that commonly exhibit strong responses in Atlantic salmon [17].

### Genes involved in adaptive immune responses

#### Attraction of B-cells and T-cells

**Fig 5:**
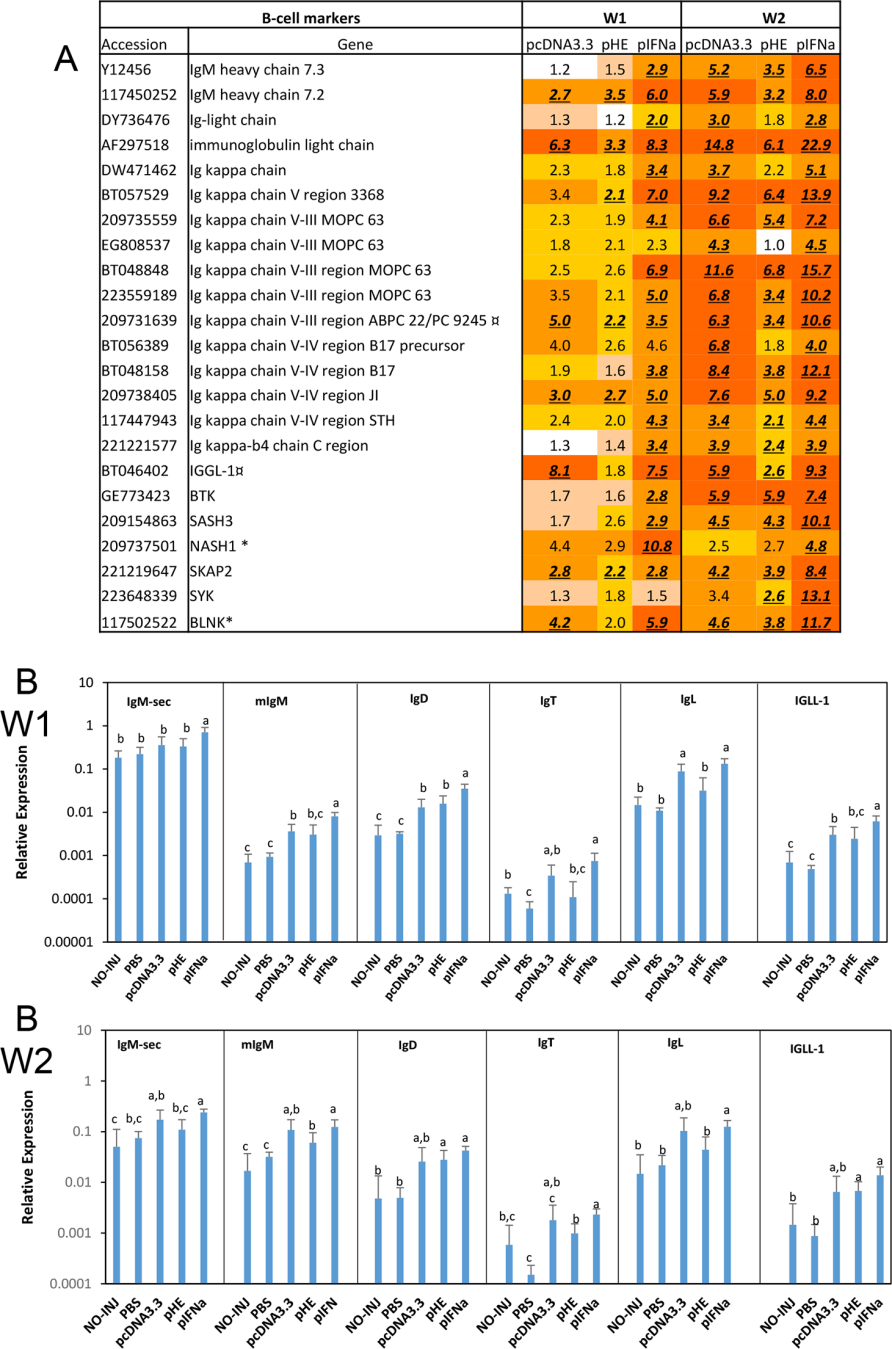
Expression of B cell marker genes. A. Microarray results. Data produced and presented as explained for Fig 3. B. Expression of immunoglobulins in response to plasmids measured by qPCR. Treatment groups and sampling for RNA extraction as described in Fig 3. Data are presented as mean gene expression relative to expression of EF1αβ +/- SD, bars not sharing common letter are significantly different p ≤ 0.05).

B-cells play an important role not only in antibody production, but also in antigen presentation. The most important markers of B-cells are immunoglobulins (Ig). The microarray data showed enhanced expression of IgM heavy chain and Ig light chains in response to all three plasmids, where the magnitude of response decreased in the range pIFNa>pcDNA3.3>pHE (Fig 5A). The qPCR results showed significant up-regulation of IgM isoforms, IgD, IgT and Ig light chain in the pIFNa group compared to PBS at both time-points (Fig 5B). The difference between pIFN and pHE was also significant at both time points except for IgD at W2. The difference between pIFNa and pcDNA3.3 was statistically significant for all genes at W1 except for IgT and Ig light chain, while at W2 there were no significant differences between the two groups. Expression of several of the Igs was higher in response to pcDNA3.3 than to pHE, but the difference was statistically significant only for Ig light chain (Fig 5B). Interestingly, both the microarray and the qPCR data showed that pcDNA3.3 and pIFNa enhanced strong and similar expression of the preB cell receptor Ig lambda-like polypeptide 1 (IGLL1). While the microarray data showed that pHE only caused a minor up-regulation of this gene (Fig 5A), the qPCR data showed no significant difference in IGLL1 expression between pcDNA3.3 and pHE (Fig 5B). Microarrays also showed that the plasmids increased abundance of transcripts from a suite of genes that control differentiation and signalling of B-cells including tyrosine kinase BTK, tyrosine kinase SYK, hematopoietic adaptor protein NASH1 and B-cell linker protein BLNK (Fig 5A).

**Fig 6:**
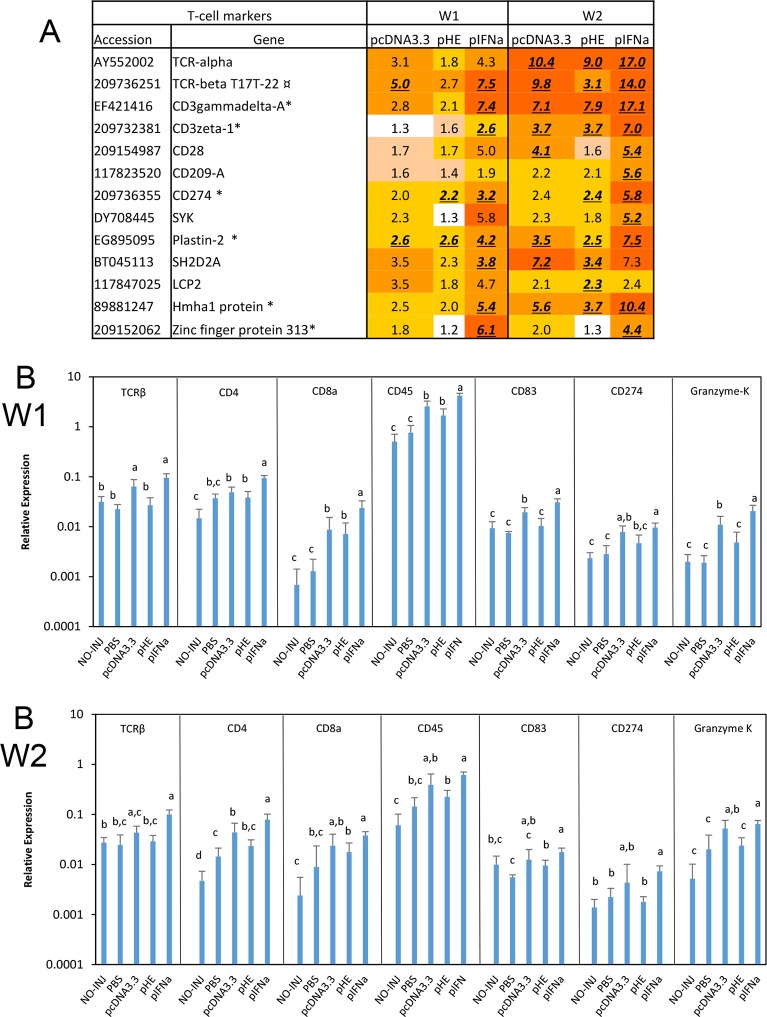
Expression of T cell marker genes. A. Microarray results. Data produced and presented as explained for Fig 3. B. Expression of T-cell markers in response to plasmids measured by qPCR. Treatment groups and sampling for RNA extraction as described in Fig 3. Data are presented as mean gene expression relative to expression of EF1αβ +/- SD, bars not sharing common letter are significantly different p ≤ 0.05).

T-cells include CD4^+^ helper cells (Th1) and CD8^+^ cytotoxic cells (CTL), which play key roles in humoral and cell mediated adaptive immunity, respectively. The common markers for T-cells are T cell receptors (TCR) alpha and TCRbeta chains, while their co-receptors CD4 and CD8 are major markers for helper and cytotoxic T cells respectively. CTLs also produce granzyme and perforin while both cell types produce IFNg. The TCR is associated with CD3epsilon, CD3delta, CD3gamma and CD3zeta (CD247) molecules in both cell types. Teleost fish possess CD3gammadelta, which is assumed to be the ancestor of CD3delta and CD3gamma [18]. Most T-cells also contain CD28, which is important for T-cell activation by interacting CD80/CD86 on APCs. The microarray data showed a strong stimulatory effect of pIFN on expression of the TCRalpha and TCRbeta chains, particularly at W2 (Fig 6A). Responses were lower to pcDNA3.3 compared to pIFNa. In general, the strongest responses were detected in the pIFNa and pcDNA3.3 groups, while pHE gave the weakest responses. Expression of other T-cell markers (CD3, CD28, CD274, SYK, Plastin, SH2D2A) followed a similar pattern of response to plasmids with pIFNa>pcDNA3.3>pHE (Fig 6A). qPCR analysis showed the same expression profile as the microarray analysis (Fig 6B). pIFNa showed the strongest response and increased the expression of TCRbeta, CD274, CD4, CD8a, CD45, CD83 and Granzyme K compared to PBS at both time points. pcDNA3.3 and pHE also augmented expression of these genes, and again the effect of pcDNA3.3 was stronger than pHE. This difference was statistically significant for TCRβ, CD83 at W1, and for Granzyme K at both time points. Taken together, the microarray and qPCR data showed that the plasmids caused enhanced expression of marker genes for B-cells and T-cells, which indicated attraction of lymphocytes to the injection sites. pIFNa caused highest expression of lymphocyte markers while pHE caused lowest expression.

#### Antigen presentation

Immune cells such as dendritic cells, B cells and macrophages are involved in antigen presentation. This process includes uptake/infection, proteasomal or endosomal degradation and presentation of antigen peptides by MHC-I or MHC-II molecules on the cell surface. MHC-I present antigenic peptides to cytotoxic T-cells while MHC-II present peptides to T-helper cells. Most cells express MHC-I, which is up-regulated by IFN due to the presence of ISRE elements in the promoter [19]. In contrast, MHC-II is expressed constitutively in antigen-presenting cells (APCs), though may be up-regulated by IFNg in some other cell types [20, 21]. Presentation of antigen peptides by MHC-I occurs in infected cells after digestion of the antigen by the immunoproteasome, transportation of peptides into ER by the TAP glycoprotein followed by loading antigenic peptides onto MHC-I molecules mediated by tapasin and other chaperone molecules [22]. APCs use MHC-II to present peptide fragments from extracellular antigens, which are taken up by endocytosis or exocytosis and are digested in endosomes.

**Fig 7:**
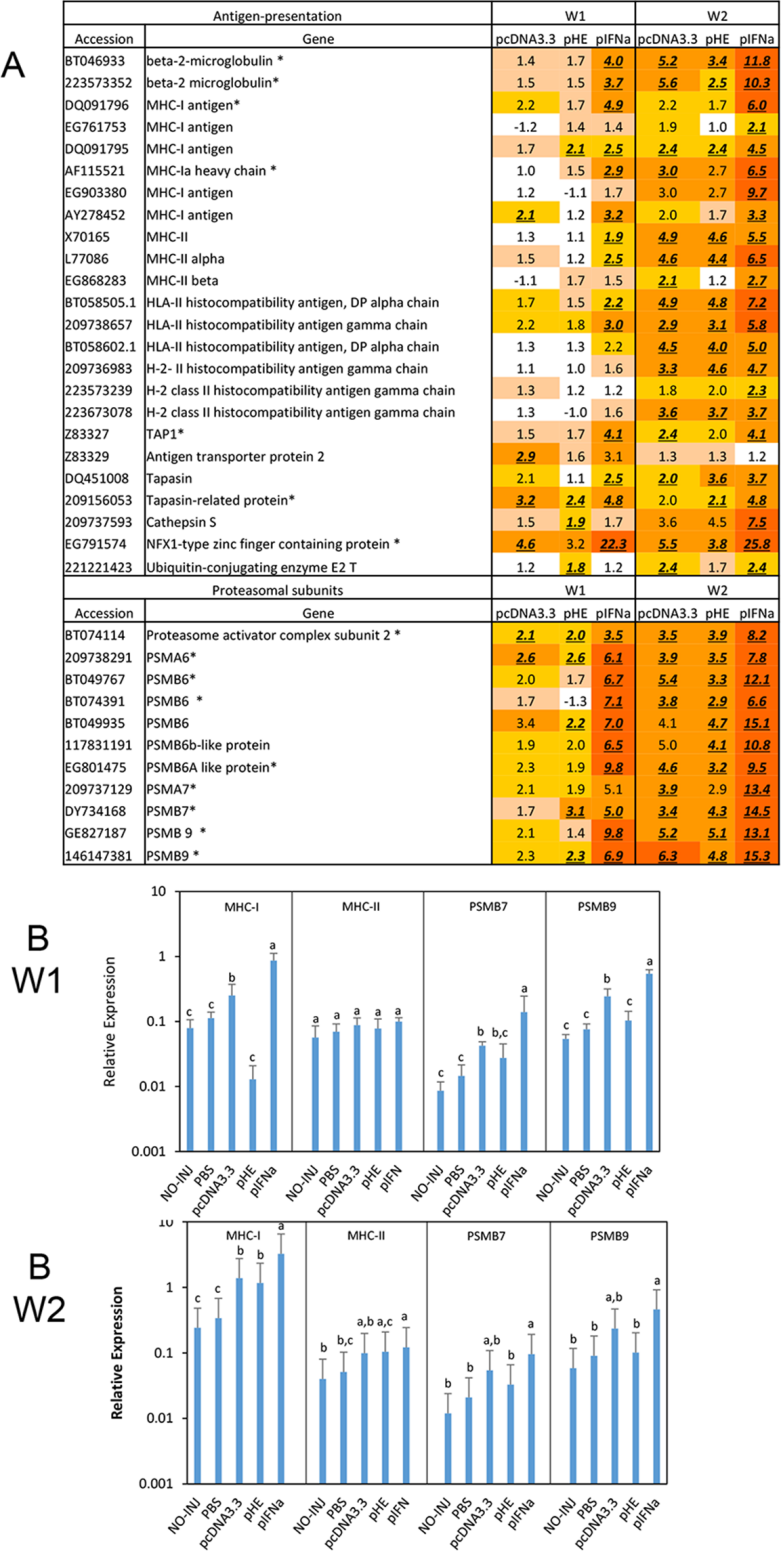
Expression of genes involved in antigen presentation. A. Microarray results. Data produced and presented as explained for Fig 3. B. Expression of genes involved in antigen presentation in response to plasmids measured by qPCR. Treatment groups and sampling for RNA extraction as described in Fig 3. Data are presented as mean gene expression relative to expression of EF1αβ +/- SD, bars not sharing common letter are significantly different p ≤ 0.05).

In the microarray study, all three plasmids up-regulated expression of genes associated with the MHC-I pathway including MHC-I antigen, beta-microglobulin, TAP and genes related to the proteasome complex with strongest stimulation at W2 (Fig 7A). pIFNa had the strongest stimulatory effect at both time points while the difference between pcDNA3.3 and pHE was minimal. The above pattern of expression was confirmed by qPCR of MHC-I, PSMB7 and PSMB9 except that pcDNA3.3 showed higher levels of expression for these genes compared to pHE (Fig 7B). The difference in expression was significant for MHC-I and PSMB9, which thus seemed to be inhibited by pHE. The plasmids also caused up-regulation of MHC-II with the highest responses at W2, which was significant for pIFNa compared to PBS. However, neither microarray nor qPCR analyses showed significant differences in expression of MHC-II between the plasmids.

### Inhibition of IFNa signalling by pHE studied by Mx reporter assay

**Fig 8:**
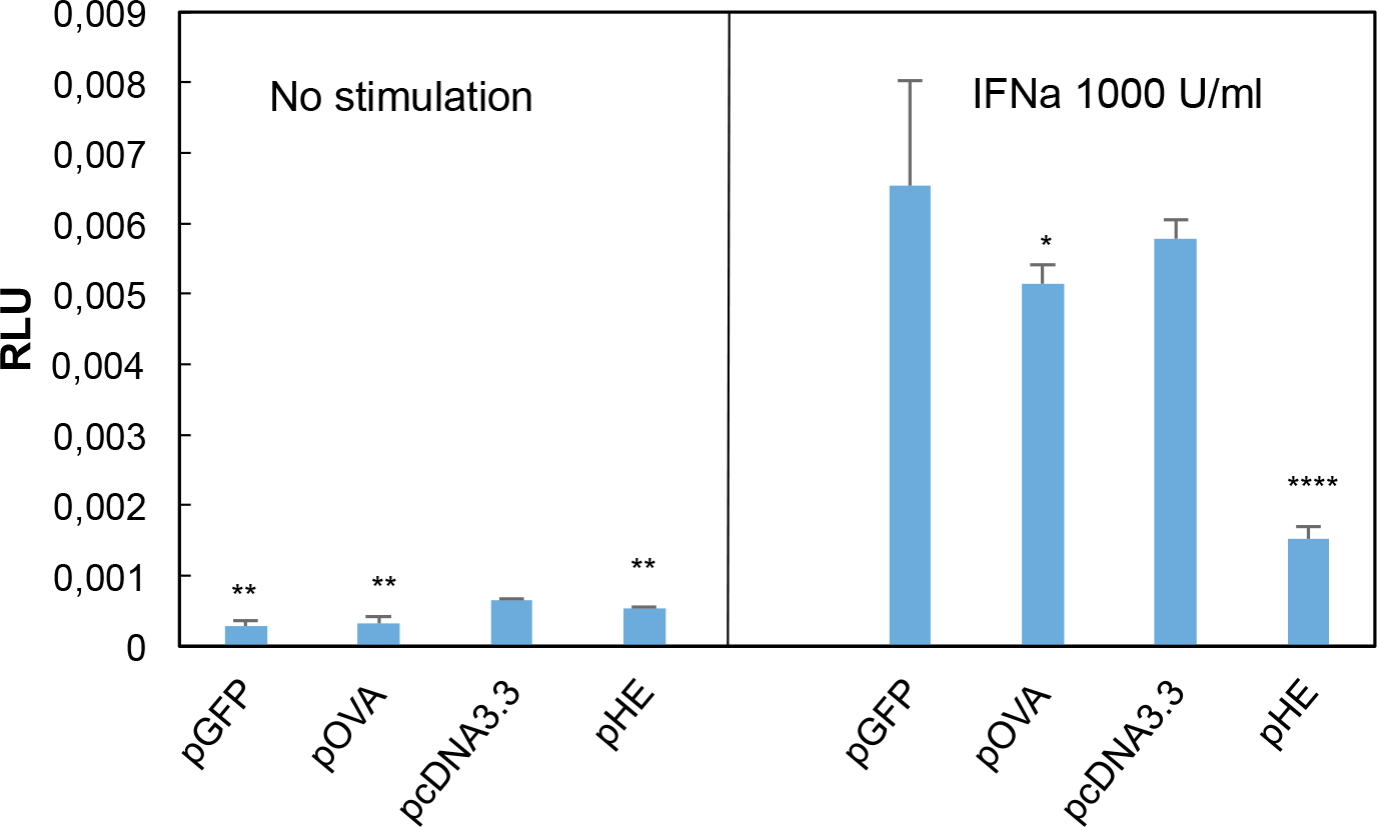
Effect on IFNa signaling of plasmids expressing HE, ovalbumin or EGFP measured by Mx reporter assay. CHSE cells were transfected with three expression constructs, a plasmid containing the Mx1 promoter fused to the firefly luciferase gene; a plasmid expressing either HE (pHE), ovalbumin (pOVA) or EGFP (pEGFP) or pcDNA3.3 (vector control); and a plasmid expressing the Renilla luciferase gene as a transfection control. Twentyfour hours after transfection, 3 wells from each treatment were stimulated for 24 hours with 1000 U/ml IFNa1 (B) and 3 wells were left untreated (A). Cells were then harvested and measured for luciferase activity. Results are presented as mean Relative Light Units (RLU) calculated by dividing blanked firefly luciferase values to blanked Renilla luciferase. Significant differences from treatment with pcDNA3.3 are indicated by * (p<0.05), ** (p<0.01), ***(p<0.001), ****(p<0.0001).

ISGs typically possess ISRE motifs in their promoters as described for Mx, ISG15 and viperin [23–25]. Both the microarray and qPCR analyses showed a stimulatory effect of pcDNA3.3 on the interferon system while the effect of pHE was lower. A reporter assay based on salmon Mx1 promoter was used to examine the effect of these plasmids on activation of the Mx1 promoter (Fig 8). Effects of plasmids expressing ovalbumin (pOVA) and EGFP (pEGFP) were also studied to examine if expression of foreign proteins in general would influence IFN signalling. CHSE 214 cells were transfected with three expression constructs, a plasmid containing the Mx1 promoter fused to the firefly luciferase gene; pHE, pOVA, pEGFP or pcDNA3.3; and a plasmid expressing the Renilla luciferase gene as a transfection control. Twenty-four hours later, three wells from each treatment were stimulated with 1000 U/ml IFNa and three wells were left without stimulation. All cells were harvested 24 h after stimulation and analysed for luciferase activity (Fig 8). The data showed similar Mx promoter activity in cells transfected with pcDNA3.3 and pEGFP and slightly lower activity in pOVA treated cells. In contrast, the Mx promoter activity was 3.8-fold lower in pHE treated cells than in pcDNA3.3 treated cells. Since pHE has a pcDNA3.3 backbone, the result indicates an inhibitory effect of HE expression on Mx promoter activity.

## Discussion

The present gene expression analyses were conducted to throw light on the adjuvant mechanisms of pIFNa in the DNA vaccine against ISAV in Atlantic salmon. The results from both microarrays and qPCR demonstrated that pIFNa mediates an increase in gene transcripts encoding proteins of four major functional types in the muscle: typical IFN induced proteins (ISGs), which are important in innate antiviral immunity; certain chemokines; markers of B- and T-lymphocytes attracted to the site of injection and proteins involved in antigen presentation. The results thus confirmed and extended qPCR results from our previous study [1]. Changes of transcripts abundance detected with microarrays and qPCR takes place through several processes: responses to plasmid DNA, responses to IFNa transcribed from pIFNa, transcripts of attracted leukocytes and modulation of these processes by HE in cells that have taken up pHE. As discussed below, attraction of lymphocytes is likely caused by the induction of chemokines by IFNa and plasmid DNA. The action of IFNa is most likely mainly accomplished by signalling through the Jak/STAT pathway. This results in activation of ISGs, which possess ISRE elements in their promoters such as the antiviral genes Mx, ISG15 and viperin [23–25]. The present study revealed that pIFNa induced these and a multitude of other genes involved in innate antiviral immunity. Chemokines may, however, be induced by other mechanisms than ISGs as discussed below for CXCL10. In mammals, adjuvant activity of type I IFNs has been explained by its direct stimulatory effect on B-cells, T-cells and DCs [2–4]. The adjuvant effect of pIFNa in salmon may thus have a dual effect, firstly by attracting lymphocytes and secondly by contributing to their activation.

Two other important findings were made in this work. The first was that both the control plasmid pcDNA3.3 and pHE enhanced transcript levels of similar genes as pIFN, but to a lower extent. Plasmid DNA may thus up-regulate these genes directly or via IFNa. pcDNA3.3 did not induce IFNa, IFNb or IFNc at W1, but did induce IFNa at W2. However, plasmid DNA may also induce IFNs at earlier time points after injection, but this was not measured. The plasmid pcDNA3.1 was found to have similar effects on transcription of IFNa and ISGs in the muscle of rainbow trout [7]. Taken together, plasmid DNA may by itself have adjuvant activity as observed in mammalian models [26, 27].

The other major finding was that pHE had a lower effect on expression of many immune genes including ISGs and chemokines compared to pcDNA3.3, which suggests that expression of HE inhibited induction of ISGs by plasmid DNA. This hypothesis was supported by the Mx-reporter assay, which showed that Mx promoter activity was significantly lower in cells transfected with pHE than in cells transfected with pcDNA3.3 alone or cells transfected with plasmids expressing ovalbumin or EGFP (Fig 8). Whether the inhibition is caused by overexpression of HE or by antagonistic activity of HE on IFN signalling, needs to be examined in future studies. It can, however, not be excluded that HE also inhibits IFN induction. Taken together, the present study suggests that the main role of pIFNa as adjuvant in the DNA vaccine against ISAV is that it has a much stronger ability to attract and stimulate immune cells at the injection site than pHE alone.

### Attraction of B- and T-cells and the role of chemokines

All three plasmids increased transcripts for markers of B-cells (IgM, Ig light chain) and T-cells (TCR, CD8) as discussed in more detail below. For several of these genes, the effect of pIFNa was higher than pcDNA3.3 at W1, while for some of the genes the differences levelled off at W2. This supports that plasmid DNA induces type I IFN, which affects genes at W2. Notably, pHE showed lower enhancement of B- and T-cell markers than pcDNA3.3, especially at W2. Accordingly, pHE does thus not seem to attract lymphocytes at the same level as pIFN or even pcDNA3.3.

An increase in transcripts of secreted IgM was observed in all three plasmid groups, which suggests attraction of B-cells that produce natural antibodies. Such cells are associated with the early innate immune response to pathogens. All three plasmids gave increased transcripts of membrane-bound IgM (mIgM), which suggests increased attraction of B-cells associated with adaptive immunity. mIgM expression was highest at W2, indicating increase with time after injection. Expression of mIgM was significantly lower in response to pHE compared to pcDNA3.3 at W2.

Attracted T-cells are likely to be a mixture of helper T-cells and CTLs as seen by increased transcripts of CD4, CD8 and granzyme K. However, CD4 and CD8 are also expressed in other cell types. A number of other genes associated with leukocytes were increased by injection of pIFNa, but the data were not sufficient for identification of other cell types.

Attraction of lymphocytes was likely due to induction of chemokines by plasmid DNA and IFNa. All three plasmids significantly increased transcripts of chemokines homologous to mammalian CCL5, CCL8 and CCL19, and chemokine receptors homologous to mammalian CCR3, CCR7, CCR9 and CXCR3. While pIFNa had the strongest effect, the differences were not significant between the pcDNA3.3 and pHE groups. The salmon CCL5-like genes belong to the macrophage inflammatory protein (MIP) group of CC-chemokines, CCL8 belongs to the monocyte chemotactic protein (MCP) group and CCL19 belongs to the CCL19/21/25 group [28]. In mammals, CCL5 has specificity for CCR1 and CCR5 and attracts T-cells and monocytes [29]. Salmon CCL5s phylogenetically group with the rainbow trout chemokine CK5B, which has been shown to attract B-cells [28, 30]. Mammalian CCL8 has specificity for CCR1, CCR2 and CCR5 and attracts many different immune cells [28, 31, 32]. Mammalian CCL19 is a ligand for CCR7, which primarily promotes homing of T cells and DCs to T cell areas of lymphoid tissues where T cell priming occurs [33]. Interestingly, rainbow trout CCR7 is present on a subpopulation of IgD^+^IgM^-^ B-cells, which indicates that salmon CCL19 also may attract such cells [34].

The qPCR data showed up-regulation of CXCL10 by all three plasmids at W1. CXCL10 is strongly induced by IFNg both in mammals and Atlantic salmon [35, 36]. In contrast to recombinant IFNg, recombinant IFNa had little effect on transcription of CXCL10 in Atlantic salmon TO cells [36]. Increased levels of CXCL10-like transcripts at the injection site may thus result from increased synthesis of IFNg either due to induction by plasmid DNA or IFNa or due to attracted IFNg expressing cells. The only cellular receptor for CXCL10 identified to date is CXCR3, which is associated with CD4^+^ Type -1 helper (Th1) and CD8^+^ cytotoxic T-cells (CTLs), but is also expressed on natural killer cells, plasmacytoid dendritic cells and subsets of B-cells [35]. However, in the present work we did not observe increased expression of CXCR3 in response to the plasmids.

A recent study identified 48 chemokine-receptors in Atlantic salmon, but their ligand specificities are not yet known [37]. However, salmon CCR7, CCR9 and CXCR4 showed unambiguous homology with their human counterparts while homology between salmon and mammalian CCR3 were less convincing. In mammals, CCR3 is a receptor for multiple chemokines and is expressed on eosinophils, basophiles, mast cells, platelets and Th2 cells; CCR7 is a receptor for CCL19 and CCL21 and is expressed on T-cells and dendritic cells; CCR9 is a receptor for CCL25 and is selectively expressed on T-cells in the thymus and small intestine [32, 38, 39].

Taken together, injection of plasmids might attract T-cells due to induction of CXCL10- and CCL19-like chemokines. It is as yet uncertain which chemokines that cause attraction of B-cells. However, pIFNa induced CCL5, which is related to trout chemokine CK5B that showed attraction for B-cells [30].

### Antigen presentation

Activation of Th1 and CTLs are dependent on presentation of peptide fragments by MHC II and MHC I, respectively. This normally occurs in professional antigen presenting cells (APCs), but it has been proposed that muscle cells also might conduct these tasks [9, 40]. The present study showed that pIFNa increased MHC I transcription, which is in agreement with the effect of recombinant IFNa on Atlantic salmon TO cells [15]. IFNa-mediated increase of MHC-I transcription is likely due to the presence of ISRE elements in the MHC-I promoter [41]. The IFNa mediated increase in MHC-I transcription was accompanied by an increase in transcripts of several members in the MHC-I antigen presentation pathway such as tapasin, TAP and members of the immunoproteasome complex (PSMB6-PSMB9) (Fig 7). It remains unknown whether this occurred in muscle cells or professional APCs. Both pcDNA3.3 and pHE also up-regulated these genes and the response was higher at W2 compared to W1. The qPCR data showed that pHE seemed to have down-regulated expression of MHC-I and PSMB9 at W1 (Fig 7B).

In contrast to MHC-I, MHC-II lacks IFN-I stimulatory elements in its promoter and is typically expressed constitutively on professional APCs [42]. The microarray analyses showed that all three plasmids gave an increase in MHC-II transcripts at W2 where pIFNa gave the strongest response. This suggests that IFNa mediates attraction of APCs. The increase in MHC-II transcripts was less clear in the qPCR analyses. Whether this is due to allelic variations is not known at the present.

### Induction of genes by pIFNa compared to DNA vaccines against fish rhabdoviruses

The DNA vaccines against the salmonid rhabdoviruses VHSV and IHNV are plasmids expressing the virus G-protein as antigen and have both been shown to induce IFNa and/or ISGs in the muscle [7, 43]. Accordingly, IFNa has been suspected to play a role in the superior protective effect of these vaccines and the present work supports this idea. The plasmid expressing the IHNV G protein (pIHNw-G) strongly augmented transcripts of genes associated with adaptive immunity similar to pIFNa in the present study [7]. This suggests that pIFNa and pIHNw-G have in common the ability to attract B- and T-cells to the injection site and to increase MHC-I mediated presentation of antigen. More recently, the DNA vaccine against VHSV based on the G-protein as antigen (pVHSV) was shown to promote recruitment of B cells to the muscle injection site [30]. Recruitment of lymphocytes to the vaccine injection site is associated with induction of chemokines both by pIFNa and the DNA vaccines against IHNV and VHSV. Common for all three plasmids is that they induce chemokines homologous to the mammalian CXCL9, CXCL10 and CXCL11, which are all induced by IFNg. However, while pIFNa most strongly induced a CXCL10-like chemokine, pVHSV and pIHNw-G induced a CXCL11-like chemokine [7, 30]. In addition, pIFNa was found to induce CCL8- and CCL19- like genes, which apparently has not been reported in the VHSV/IHNV DNA vaccine studies. In the VHSV DNA vaccine work, expression of 12 different chemokines was studied [30]. In addition to the CXCL11-like chemokine, pVHSV was only found to enhance transcripts of the chemokine CK5B, which groups with mammalian CCL4/CCL5 and the chemokine CK6, which groups with mammalian CCL17/CCL22 [30]. Trout CK5B and CK6 showed chemotactic properties for B-cells and were suggested to cause recruitment of B-cells to the injection site of the VHSV DNA vaccine [30]. In the present study, pIFNa was found to enhance expression of a CCL5-like protein, which showed strong homology with trout CK5B. On the other hand, pIFNa had little effect on expression of the salmon CK6-like gene.

## Conclusion

The adjuvant effect of pIFNa in the DNA vaccine against ISAV appears to result from recruitment and possibly stimulation of B-cells, T-cells and antigen presenting cells. Plasmid DNA is likely to also possess adjuvant activity since pcDNA3.3 increased transcripts of a similar set of genes as pIFNa, but at a lower level. The finding that pHE showed lower enhancement of ISGs and chemokine expression than pcDNA3.3, suggests that expression of HE inhibits induction of genes induced by plasmid DNA. A main function of pIFNa as adjuvant in the DNA vaccine against ISAV may thus be to overcome the inhibitory action of HE on expression of ISGs.

## Supporting information

S1 Table List of primers used in qPCR. (DOCX)

S2 Table Mean fold change in expression of genes in muscle at the injection site of plasmids measured by qPCR. Transcripts levels were normalized against EF1αβ and the results are presented as fold change relative to the PBS group. Treatment groups and sampling for RNA extraction as described in Fig 3.
(DOCX)

S1 Fig Expression of genes involved in IFN induction and IFN signalling. A. Microarray data of IRFs, STATs and JAK1. Data produced and presented as explained for Fig 3.
B. Expression of IRFs and STATs in response to plasmids measured by qPCR. Treatment groups and sampling for RNA extraction as described in Fig 3. Data are presented as mean gene expression relative to expression of EF1αβ +/- SD, bars not sharing common letter are significantly different (p ≤ 0.05).
(PDF)
