# Supplementary material for: Transcriptome analysis of plasmid-induced genes sheds light on the role of type I IFN as adjuvant in DNA vaccine against infectious salmon anemia virus
Source: PLoS One. 2017 Nov 21;12(11):e0188456. doi: 10.1371/journal.pone.0188456 (PMC5697855; doi:10.1371/journal.pone.0188456)
Supplement: S1 Table — (DOCX) [file pone.0188456.s001.docx]

S1 Table. List of primers used for qPCR

| Gene | Sequence | Acc. No. |
| --- | --- | --- |
| IFNa1-Fw | TGCAGTATGCAGAGCGTGTG | DQ354152 |
| IFNa1-Rev | TCTCCTCCCATCTGGTCCAG |  |
| IFNb-Fw | GCATTGGAGGCTATGCGATAT | EU768890 |
| IFNb-Rev | TTCCCAAACACCACCTACGACA |  |
| IFNc-Fw | ATGTATGATGGGCAGTGTGG | EU768890 |
| IFNc-Rev | CCAGGCGCAGTAACTGAAAT |  |
| IFNg-Fw | AAGGGCTGTGATGTGTTTCTG | AY795563 |
| IFNg-Rev | TGTACTGAGCGGCATTACT |  |
| Mx1-Fw | TGCAACCACAGAGGCTTTGAA | U66475 |
| Mx1-Rev | GGCTTGGTCAGGATGCCTAAT |  |
| ISG15-Fw | CTGAAAAACGAAAAGGGCCA | AY926456 |
| ISG15-Rev | GCAGGGACTCCCTCCTTGTT |  |
| Viperin-Fw | TCCTTGATGTTGGCGTGGAA | BT047610 |
| Viperin-Rev | GCATGTCAGCTTTGCTCCACA |  |
| IRF1-Fw | GCAATGAAGTAGGCACAGCA | BT048538 |
| IRF1-Rev | CGCAGCTCTATTTCCGTTTC |  |
| IRF3-Fw | GGCCAAACTGACCCAAGATA | BT059292 |
| IRF3-Rev | GCCTCCTGCTCTTGTCAAAC |  |
| IRF7-Fw | CCAGTGCCACCAGTCCTAAT | NM_001136548 |
| IRF7-Rev | GGTGATCTCCAAGTCCCAGA |  |
| STAT1-Fw | GTTCAGGATGCAGAGCATGA | BT073175 |
| STAT1-Rev | TGTCTGTGCTTCCACTCAGG |  |
| STAT2-Fw | TCAACATGCTTTGCCTTGAG | KJ155790 |
| STAT2-Rev | GTTTGTGTGCGATCATGTCC |  |
| STAT3-Fw | TCGGAGACCCGTGTAGTACC | XM_014192882 |
| STAT3-Rev | CTCATTGTGCATCAGCGAGT |  |
| CCL5-Fw | TCGCCGTCGTTCTCTCGTTA | XP_014047283 |
| CCL5-Rev | CCAATGGCTGCAGATGGT |  |
| CCR9-Fw | ACCAGGACAACTCCACCAAG | BT046009 |
| CCR9-Rev | GTATGGCAACTGGGAGAGGA |  |
| CCL19-Fw | TGGCTGCATTTCTTCTGTTG | XM_014133457 |
| CCL19-Rev | GCGATTTCCTCTTCAGTTGC |  |
| CXCL10-Fw | TGAAGAACGGAAAAGGGATG | EF619047 |
| CXCL10-Rev | TCAGGGCACTGACTCAACTG |  |
| CCR4-Fw | TCATGTCAACCCGAATGAGA | XM_014190881 |
| CCR4-Rev | TGGAGGGCTCTCAGCAGTAT |  |
| CCR7-Fw | AGCTTCTACAGCGGCATGTT | XM_014177670 |
| CCR7-Rev | CATCACCCAGATGACCACAG |  |
| mIgM-Fw | CCTACAAGAGGGAGACCGA | BT059185 |
| mIgM-Rev | GATGAAGGTGAAGGCTGTTTT |  |
| IgM-Sec-Fw | CTACAAGAGGGAGACCGGAG | Y12457 |
| IgM-Sec-Rev | AGGGTCACCGTATTATCACTAGTTT |  |
| IgD-Fw | GTGTCTGCAGCAGCCTATGA | AF278717 |
| IgD-Rev | GTGAAGTCCTTCCCCTGGTT |  |
| IgT-Fw | CAACACTGACTGGAACAACAAGGT | [ACX50290](http://www.ncbi.nlm.nih.gov/entrez/query.fcgi?cmd=search&db=protein&doptcmdl=genbank&term=ACX50290%5baccn%5d) |
| IgT-Rev | CGTCAGCGGTTCTGTTTTGGA |  |
| IgL-Fw | TCCCTCCATCCACTGAAGAC | BT046402 |
| IgL-Rev | AGAGAATGTTGACCCCAACG |  |
| IGLL-Fw | CTGAGCAGCAACCAGACAAA | XM_014184334 |
| IGLL-Rev | AGAGAATGTTGACCCCAACG |  |
| TCR-B-Fw | AAGACTGGCACAACCCAGAC | X97435 |
| TCR-B- Rev | CAGTCTGGGTGCTCTTCACA |  |
| CD4-Fw | GTTGAAAGGGCGAAAGTGAG | NM_001146408 |
| CD4-Rev | GTGCCTTCGATGAGGACATT |  |
| CD8a-Fw | CGTCTACAGCTGTGCATCAATCAA | NM_001123583 |
| CD8a-Rev | GGCTGTGGTCATTGGTGTAGTC |  |
| CD45-Fw | TCGTCATGCTGTCAGACCTC | XM_014123572 |
| CD45-Rev | TGCCGACTGTCTTTCCTCTT |  |
| CD83-Fw | TGCCTACCCAGGAAGTGAAG | DQ339141 |
| CD83-Rev | GAGGGCTCCTCACCTAGCTT |  |
| CD274-Fw | GATCAACGACTCTGGGGTGT | BT049246 |
| CD274-Rev | GGTGACATGGAAAAGCTGGT |  |
| Granzyme-K-Fw | CCAGGACATGCTCTGTGCTA | XM_014133293 |
| Granzyme-K-Rev | CTTTTGGAGAACCGGGTGTA |  |
| MHC-I-Fw | GAAGAGCACTCTGATGAGGACAG | JN561338 |
| MHC-I-Rev | CACCATGACTCCACTGGGGT |  |
| MHC-II-Fw | ATGGTGGAGCACATCAGCC | ABX44766 |
| MHC-II-Rev | CTCAGCCTCAGGCAGGGAC |  |
| PSMB7-Fw | GAAATGCGCGACTGTAGTGA | XM_014141746 |
| PSMB7-Rev | TGGTCCCTGTCTTCATAGGC |  |
| PSMB9-Fw | AAGTTCGTTCAGCTGCCACT | AF184936 |
| PSMB9-Rev | GTTGGCAGTCCTCTTTGCTC |  |
| EF1a-Fw | TGCCCCTCCAGGATGTCTAC | [BG933853](http://www.ncbi.nlm.nih.gov/entrez/query.fcgi?cmd=search&db=nucleotide&doptcmdl=genbank&term=BG933853) |
| EF1a-Rev | CACGGCCCACAGGTACTG |  |
