# Supplementary material for: Transcriptome analysis of plasmid-induced genes sheds light on the role of type I IFN as adjuvant in DNA vaccine against infectious salmon anemia virus
Source: PLoS One. 2017 Nov 21;12(11):e0188456. doi: 10.1371/journal.pone.0188456 (PMC5697855; doi:10.1371/journal.pone.0188456)
Supplement: S2 Table — Transcripts levels were normalized against EF1αβ and the results are presented as fold change relative to the PBS group. Treatment groups and sampling for RNA extraction as described in Fig 3. (DOCX) [file pone.0188456.s002.docx]

S2 Table. Mean fold change in expression of genes in muscle at the injection site of plasmids measured by qPCR.

|  | W1 | | | | W2 | | | |
| --- | --- | --- | --- | --- | --- | --- | --- | --- |
| Gene | **PBS** | **pcDNA3.3** | **pHE** | **pIFNa** | **PBS** | **pcDNA3.3** | **pHE** | **pIFNa** |
| IFNa | 1.01 | 3.00 | 2.14 | 114.02 | 1.18 | 27.94 | 1.59 | 72.23 |
| IFNb | 1.03 | 2.78 | 0.99 | 13.03 | 1.16 | 4.48 | 2.27 | 4.17 |
| IFNc | 1.02 | 1.79 | 1.07 | 1.61 | 1.04 | 1.44 | 0.70 | 1.46 |
| IFNγ | 1.05 | 11.67 | 6.57 | 22.80 | 0.95 | 0.71 | 1.59 | 6.83 |
| Mx1 | 1.10 | 15.51 | 5.00 | 86.44 | 1.34 | 11.97 | 4.82 | 42.93 |
| Viperin | 1.12 | 25.32 | 7.38 | 118.47 | 1.03 | 18.97 | 0.52 | 15.06 |
| ISG15 | 1.17 | 60.90 | 18.26 | 223.83 | 0.99 | 0.30 | 0.72 | 26.89 |
| IRF1 | 1.03 | 5.41 | 3.43 | 8.21 | 1.05 | 5.39 | 3.04 | 7.03 |
| IRF3 | 1.05 | 7.08 | 1.79 | 12.50 | 1.07 | 3.96 | 1.21 | 8.31 |
| IRF7 | 1.02 | 7.34 | 2.74 | 14.82 | 1.11 | 7.19 | 1.94 | 18.60 |
| STAT1 | 1.19 | 8.21 | 1.98 | 56.37 | 1.15 | 5.82 | 2.46 | 19.17 |
| STAT3 | 1.00 | 3.52 | 1.30 | 5.57 | 1.07 | 2.23 | 0.80 | 4.14 |
| STAT7 | 0.93 | 1.29 | 0.83 | 0.96 | 1.01 | 1.64 | 1.09 | 1.13 |
| CCL5 | 1.58 | 4.93 | 4.46 | 20.45 | 1.23 | 34.18 | 5.78 | 41.93 |
| CCL19 | 1.02 | 2.26 | 0.92 | 5.54 | 1.43 | 6.04 | 2.40 | 10.27 |
| CxCL10 | 1.10 | 64.96 | 31.28 | 99.95 | 1.19 | 33.90 | 8.88 | 101.57 |
| CCR4 | 1.29 | 1.58 | 1.07 | 2.02 | 0.87 | 1.53 | 1.91 | 1.65 |
| CCR7 | 1.06 | 4.25 | 4.76 | 11.26 | 1.18 | 6.74 | 5.48 | 9.55 |
| CCR9 | 1.09 | 5.34 | 2.58 | 15.91 | 1.43 | 6.04 | 2.40 | 10.27 |
| IgM-sec | 1.15 | 1.87 | 1.74 | 3.72 | 1.05 | 2.43 | 1.54 | 3.37 |
| mIgM | 1.02 | 3.94 | 3.34 | 8.82 | 1.02 | 3.46 | 1.95 | 3.99 |
| IgD | 1.01 | 4.13 | 5.02 | 11.17 | 1.21 | 6.29 | 6.83 | 10.35 |
| IgT | 1.08 | 6.22 | 1.98 | 13.49 | 1.12 | 13.34 | 7.28 | 17.15 |
| IgL | 1.01 | 8.18 | 2.95 | 12.29 | 1.11 | 5.22 | 2.23 | 6.37 |
| IGLL-1 | 1.02 | 6.40 | 5.16 | 13.11 | 1.27 | 9.38 | 9.85 | 20.11 |
| TCRβ | 1.02 | 2.88 | 1.22 | 4.31 | 1.11 | 1.94 | 1.31 | 4.49 |
| CD4 | 1.02 | 1.35 | 1.06 | 2.61 | 1.08 | 3.25 | 1.74 | 5.83 |
| CD8 | 1.32 | 8.92 | 7.36 | 24.35 | 1.09 | 5.66 | 4.24 | 9.08 |
| CD45 | 0.57 | 1.94 | 1.28 | 3.16 | 1.09 | 2.96 | 1.70 | 4.69 |
| CD83 | 0.85 | 2.22 | 1.18 | 3.51 | 1.01 | 2.27 | 1.73 | 3.23 |
| CD274 | 1.07 | 2.97 | 1.77 | 3.64 | 1.10 | 2.14 | 0.89 | 3.60 |
| Granzyme | 1.05 | 6.08 | 2.67 | 11.42 | 1.31 | 3.42 | 1.57 | 4.21 |
| MHC-I | 1.04 | 2.74 | 0.13 | 8.14 | 1.78 | 6.04 | 5.30 | 14.66 |
| MHC-II | 1.07 | 1.35 | 1.20 | 1.60 | 1.02 | 1.78 | 1.97 | 2.72 |
| PSMB7 | 1.10 | 3.20 | 2.08 | 10.58 | 1.06 | 2.75 | 1.67 | 4.86 |
| PSMB9 | 1.02 | 3.27 | 1.38 | 7.30 | 1.06 | 2.74 | 1.18 | 5.38 |
