# Supplementary material for: Transcriptome analysis of plasmid-induced genes sheds light on the role of type I IFN as adjuvant in DNA vaccine against infectious salmon anemia virus
Source: PLoS One. 2017 Nov 21;12(11):e0188456. doi: 10.1371/journal.pone.0188456 (PMC5697855; doi:10.1371/journal.pone.0188456)
Supplement: S1 Fig — A. Microarray data of IRFs, STATs and JAK1. Data produced and presented as explained for Fig 3. B. Expression of IRFs and STATs in response to plasmids measured by qPCR. Treatment groups and sampling for RNA extraction as described in Fig 3. Data are presented as mean gene expression relative to expression of EF1αβ +/- SD, bars not sharing common letter are significantly different (p ≤ 0.05). (PDF) [file pone.0188456.s003.pdf]

A

Genes associated with IFN induction and IFN signaling		W1			W2		
Accession	Gene	pcDNA3.3	pHE	pIFNa	pcDNA3.3	pHE	pIFNa
209734939	IRF 1*	***3.5***	***2.7***	***6.2***	***6.3***	***6.4***	***20.5***
209151224	IRF 2*	1.2	-1.0	***3.3***	***3.3***	***2.3***	***7.1***
223648493	IRF 3*	***2.3***	1.5	***8.6***	***3.1***	***2.9***	***7.6***
209150530	IRF 5*	1.9	1.2	***3.7***	2.3	1.7	***5.6***
DY725939	IRF 7*	3.5	***2.9***	6.7	1.9	-1.4	12.7
EG923013	IRF 8 *	1.9	***2.7***	***4.2***	3.7	***4.1***	***6.7***
209735717	STAT1-1*	***5.3***	2.5	***10.8***	***5.7***	***5.6***	***20.7***
209155193	STAT1-2 *	***2.4***	1.5	***5.5***	***3.6***	***2.3***	***8.0***
EG911621	STAT2*¤	***2.9***	1.7	***5.2***	***3.6***	***1.9***	***7.1***
DY741158	STAT3¤	2.7	1.3	5.1	1.9	-2.3	7.1
EG896315	STAT3-like	-1.4	-1.5	-1.3	-1.7	-1.3	***-2.3***
223649055	STAT5B	-1.3	***-2.0***	-1.6	***-1.9***	-1.6	***-2.1***
117425401	JAK1	2.9	2.1	***7.2***	2.4	2.3	***3.4***

B W1

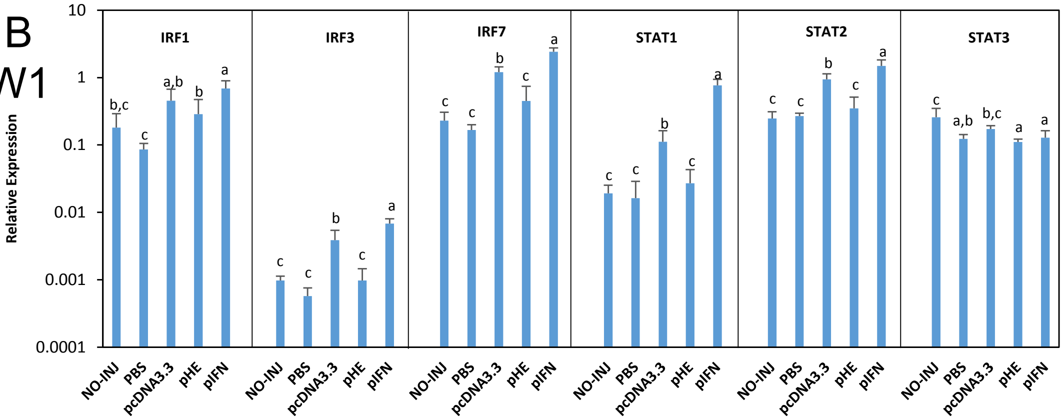

B W2

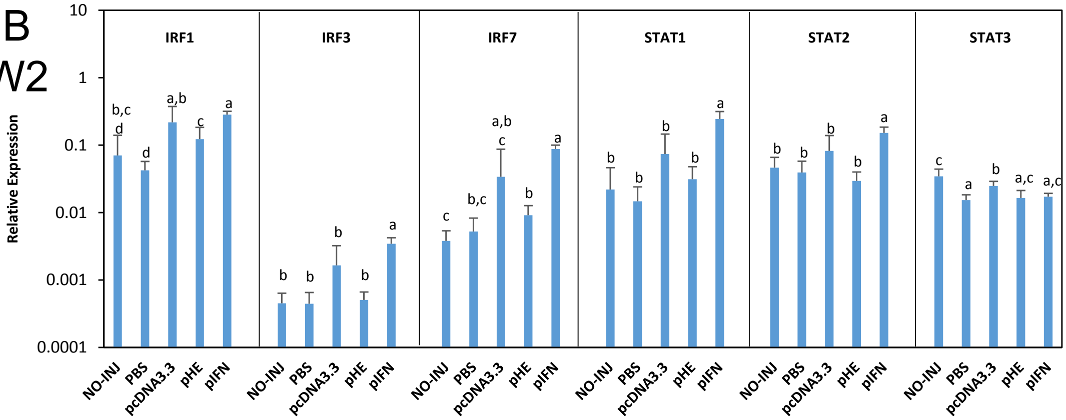

Fig. S1
